# Structural network alterations in patients with nasopharyngeal carcinoma after radiotherapy: A 1-year longitudinal study

**DOI:** 10.3389/fnins.2022.1059320

**Published:** 2022-11-17

**Authors:** Xinyuan Zhang, Jie Pan, Yuhao Lin, Gui Fu, Pu Xu, Jiahui Liang, Chenfei Ye, Jie Peng, Xiaofei Lv, Yadi Yang, Yanqiu Feng

**Affiliations:** ^1^School of Biomedical Engineering, Guangdong Provincial Key Laboratory of Medical Image Processing, Guangdong Province Engineering Laboratory for Medical Imaging and Diagnostic Technology, Southern Medical University, Guangzhou, China; ^2^Department of Medical Imaging, Sun Yat-sen University Cancer Center, State Key Laboratory of Oncology in South China, Collaborative Innovation Center for Cancer Medicine, Guangdong Key Laboratory of Nasopharyngeal Carcinoma Diagnosis and Therapy, Guangzhou, China; ^3^International Research Institute for Artificial Intelligence, Harbin Institute of Technology, Shenzhen, China; ^4^Guangdong-Hong Kong-Macao Greater Bay Area Center for Brain Science and Brain-Inspired Intelligence, Key Laboratory of Mental Health of the Ministry of Education, Southern Medical University, Guangzhou, China

**Keywords:** nasopharyngeal carcinoma, radiotherapy, radiation-induced brain injury, structural network, diffusion tensor imaging

## Abstract

This longitudinal study explored the changed patterns of structural brain network after radiotherapy (RT) in patients with nasopharyngeal carcinoma (NPC). Diffusion tensor imaging (DTI) data were gathered from 35 patients with NPC at four time points: before RT (baseline), 0∼3 (acute), 6 (early delayed), and 12 months (late-delayed) after RT. The graph theory was used to characterize the dynamic topological properties after RT and the significant changes were detected over time at the global, regional and modular levels. Significantly altered regional metrics (nodal efficiency and degree centrality) were distributed in the prefrontal, temporal, parietal, frontal, and subcortical regions. The module, that exhibited a significantly altered within-module connectivity, had a high overlap with the default mode network (DMN). In addition, the global, regional and modular metrics showed a tendency of progressive decrease at the acute and early delayed stages, and a partial/full recovery at the late-delayed stage. This changed pattern illustrated that the radiation-induced brain damage began at the acute reaction stage and were aggravated at the early-delayed stage, and then partially recovered at the late-delayed stage. Furthermore, the spearman’s correlations between the abnormal nodal metrics and temporal dose were calculated and high correlations were found at the temporal (MTG.R and HES.L), subcortical (INS.R), prefrontal (ORBinf.L and ACG.L), and parietal (IPL.R) indicating that these regions were more sensitive to dose and should be mainly considered in radiotherapy treatment plan.

## Introduction

Nasopharyngeal carcinoma (NPC) is a malignant tumor, and it is mostly found in Southern China and Southeast Asia (Chan, 2010; Tabuchi et al., 2011). Radiotherapy (RT) with or without adjuvant chemotherapy is the primary treatment for patients with NPC. However, the normal brain tissues surrounding the tumor are inevitably irradiated during cranial irradiation, causing brain abnormalities and cognitive decline. These abnormalities may compromise the quality of life of patients with NPC. Based on the pathophysiology of the side effects of RT, the time following RT can be classified into acute reaction period (days–weeks) (post-RT-AC), early-delayed period (1–6 months) (post-RT-ED), and late-delayed period (> 6 months) (post-RT-LD) (Lell, 2015). The RT-related brain changes are different during different periods but how the RT-related brain damage evolves over time is still unclear. Therefore, it is essential to further explore the temporal brain changes after completing RT which may facilitate clinical diagnosis and early intervention.

Recently, few cross-sectional or longitudinal studies have demonstrated that normal-appearing brain tissues underwent different changes at different post-RT periods in patients with NPC using various magnetic resonance imaging (MRI) analysis techniques (Lin et al., 2017, 2021; Guo et al., 2018; Lv et al., 2019; Wu et al., 2020; Qiu et al., 2021). Specifically, our previous longitudinal studies found that the volumes of the gray matter (Guo et al., 2018) and white matter (WM) in bilateral temporal subfields (Lin et al., 2021) and bilateral hippocampal subfields (Lv et al., 2019) decreased over time after RT. In addition, the cross-sectional or longitudinal studies on cortical brain morphology revealed progressive RT-induced reduction in cortical volume, cortical thickness, and cortical surface area, mainly in the temporal, basal occipital, and basal frontal lobes (Lin et al., 2017; Zhang et al., 2018). Aside brain morphological alteration, the WM microstructure changed after RT in patients with NPC (Wang et al., 2012; Xiong et al., 2013; Chen et al., 2015, 2020; Duan et al., 2016; Leng et al., 2017, 2019; Ding et al., 2018). Diffusion tensor imaging (DTI) is the only non-invasive MRI technique to assess brain white matter microstructure *in vivo* (Le Bihan et al., 2001). Most DTI studies adopted the regions of interest (ROI)-based analysis strategy to detect the microstructural changes in the temporal lobe of patients with NPC (Wang et al., 2012; Xiong et al., 2013; Chen et al., 2015). They found that diffusion metrics, such as FA and ADC in the temporal lobe, exhibited dose-related dynamic alterations over time after RT. Nevertheless, the ROI-based analysis is limited to specific regions and cannot reflect whole-brain changes. Recently, some studies investigated the changes in whole-brain WM at different post-RT periods by voxel-based analysis (Duan et al., 2016; Leng et al., 2017, 2019; Ding et al., 2018). They found that RT-induced brain alterations were dynamic and extensive, and were not limited to the temporal lobe.

However, the voxel-based analysis cannot reflect the dynamic interaction of distinct brain regions. The graph theory analysis models brain connectivity as a network to assess the structural and functional brain organization (Sporns, 2011), offering an opportunity to better understand how the brain changes from a network perspective. The structural connectivity (SC) network is usually considered to be the physical substrate of the functional connectivity (FC) network. In patients with NPC, functional and structural brain network topology change after RT (Ma et al., 2016; Tian and Zhao, 2017; Qiu et al., 2018; Leng et al., 2019; Chen et al., 2020). For structural brain networks, a longitudinal DTI study reported that both global and local efficiencies, as well as the nodal topology, were altered in post-RT patients (Tian and Zhao, 2017). This study only investigated the difference between pre-RT and post-RT, but did not consider the different patterns of brain changes at different post-RT periods. Subsequently, a cross-sectional DTI study on three points (baseline, post-RT-ED, and post-RT-LD) found that structural topological properties were altered in the post-RT-ED but began recovering in the post-RT-LD (Chen et al., 2020). Nevertheless, in this cross-sectional study, the data with different post-RT durations were not from the same group of patients with NPC; the cohort effect could compromise the ability to detect the RT-induced brain alteration; the study did not investigate the acute reaction period which exhibits different side effect of RT when compared to the post-RT-ED and post-RT-LD periods. Inclusion of three post-RT periods will facilitate better understand the RT-related brain changed patterns over time.

Therefore, this work will adopt a longitudinal study with four time points (baseline, post-RT-AC, post-RT-ED, and post-RT-LD) to investigate the dynamic changes in structural brain network. Our cohort group included 35 patients with NPC, and each patient was followed up with four repeated scans: prior to RT, 0∼3, 6, and 12 months follow-up after the completion of RT. The topological properties of the structural network at the global, regional, and modular levels were calculated. Based on the analysis of these topological properties, the dynamic brain changes after RT and the relationship between these brain alterations and radiation dose were assessed.

## Materials and methods

### Patients

Forty-three newly diagnosed treatment-naïve patients with NPC (aged 18–60) were initially enrolled. The inclusion criteria were as follows: right-handedness, no alcoholism or substance dependence, no high blood pressure, no diabetes, no brain tumors, no visible brain lesions, no history of cranial trauma, no history of any psychiatric or neurological disease, no current medications that may affect cognitive function, and no contraindications for MRI scanning. Among 43 enrolled patients, eight patients with NPC were excluded because their DTI images suffered severe geometric distortions and/or motion artifacts, which could not be corrected by the post-processing technique. Finally, 35 patients with NPC (21 males; aged 23–60 years; averaging 40.11 ± 8.88 years) were selected and analyzed in this study. This study was approved by the Institutional Review Board of the Sun Yat-sen University Cancer Center. All participants provided written informed consent.

### Treatment

All patients were treated with intensity-modulated radiotherapy (IMRT) (*n* = 32) or tomotherapy (TOMO) (*n* = 3), the details of which have been reported by previous studies (Sun et al., 2013; Tang et al., 2015). The prescribed regimen included a total dose of 68–70 Gy in 30–33 fractions at 2.12–2.33 Gy/fraction to the planning target volume (PTV) of the primary gross tumor volume (GTVnx), 60–70 Gy to the PTV of GTV of involved lymph nodes (GTVnd), 60–64 Gy to the PTV of the high-risk clinical target volume (CTV1), and 54–58 Gy to the PTV of the low-risk clinical target volume (CTV2). All patients received one fraction daily over a period of about 45 days, five consecutive days per week. Based on the guidelines defined by the 7th edition of the AJCC staging system for NPC, the patients with stage I to IIa disease received no chemotherapy, those with stage IIb received concurrent chemotherapy, and those with stages III to IVa–b received concurrent chemotherapy with/without neoadjuvant/adjuvant chemotherapy (Edge et al., 2010).

### Follow-up procedure

To assess the dynamic alterations in structural brain network topology after RT, we repeatedly performed MRI scanning at the following stages for each patient: before initiation of RT (baseline), 0∼3 months (post-RT-AC), 6 months (post-RT-ED), and 12 months (post-RT-LD) after RT. Since the MRI data at each stage were acquired from the same group (35 patients), a longitudinal comparison strategy was performed to avoid potential bias due to cohort effect.

### MRI acquisition

The MRI images were acquired on a GE Discovery MR 750 3.0T scanner (GE Medical Systems, WI, USA) at the Department of Medical Imaging, Sun Yat-sen University Cancer Center. The high-resolution T1-weighted volume data were acquired using three-dimensional spoiled gradient-recalled sequence with the following parameters: TR/TE = 8.2/3.2 ms, TI = 800 ms, flip angle = 8°, field of view = 256 × 256 × 180 mm^3^, acquisition matrix = 256 × 256 × 180, voxel size = 1 × 1 × 1 mm^3^. The DTI data were acquired using a twice-refocused spin-echo diffusion-weighted (DW) echo-planar imaging sequence with the following parameters: TR/TE = 10,000/63.8 ms, acquisition matrix = 128 × 128, field of view = 256 × 256 mm^2^, in-plane resolution = 2 × 2 mm^2^, slice thickness = 2 mm without inter-slice gap, 75 axial slices covering the whole brain, one volume with b = 0 s/mm^2^, 30 volumes with b = 1,000 s/mm^2^.

### Data preprocessing and tractography

The data preprocessing included the following steps: (1) denoising the DW images using Marchenko-Pastur PCA (Veraart et al., 2016); (2) correcting the eddy current and head motion-induced distortion with an affine transformation; (3) skull stripping for the T1-weighted images and non-DW images (*b* = 0 s/mm^2^) with FSL-Brain Extraction Tool (BET).

Whole-brain fiber reconstruction was performed for each diffusion data in native space using probabilistic tracking. Anatomically constrained tractography (Smith et al., 2012), seeding from the interface between grey matter and white matter, was used to achieve an anatomically plausible trajectory. A total of 10 million (M) seeding streamlines were initially generated and tracked. Finally, Spherical-deconvolution Informed Filtering of Tractograms (SIFT) (Smith et al., 2013) was performed to filter the streamlines from 10 to 1 M for improving the quantitative nature of whole-brain streamline reconstruction.

All these preprocessing steps and fiber tracking were accomplished within MRtrix3,^1^ which is an open-source software package and includes scripts that interface with external packages, such as FSL^2^ (Jenkinson et al., 2012).

### Structural network construction

**FIGURE 1:**
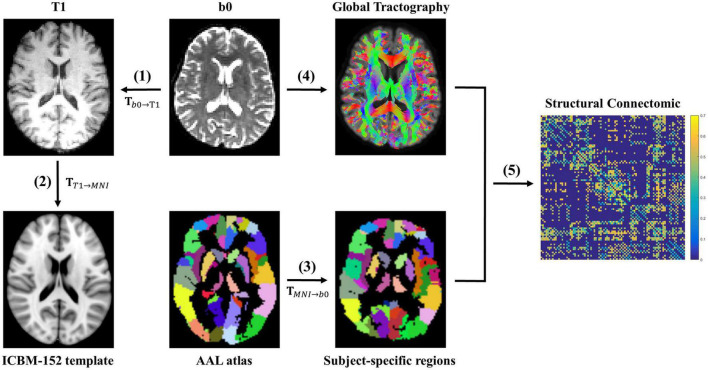
The flowchart of white matter structural network construction. **(1)** The non-diffusion image b0 was co-registered to the corresponding T1-weighted image with an affine transformation **T**_*b*0→*T*1_ for each participant. **(2)** The T1-weighted image was non-linearly transformed to the Montreal Neurological Institute (MNI) space using the ICBM-152 brain template, resulting in a non-linear transformation **T**_*T*1→*MNI*_. **(3)** These two transformations (**T**_*b*0→*T*1_ and **T**_*T*1→*MNI*_) were inversed and combined into one transformation (**T**_*MNI*→*b*0_), which was applied to wrap the AAL from the MNI space to the native diffusion space of each participant. Thereafter, 90 participant-specific brain regions were created. **(4)** A whole-brain fiber reconstruction was performed for each subject in native space using the probabilistic tracking in conjunction with DTI. **(5)** Finally, the 90 × 90 symmetric connectivity matrix for each participant was constructed by calculating the mean FA values of streamlines that connect each region pair.

Figure 1 shows the flow chart of structural network construction, which is also accomplished within MRtrix3. First, 90 brain regions (nodes) were created for each participant with the automated anatomical labeling (AAL) atlas (Tzourio-Mazoyer et al., 2002). Particularly, for each participant, the non-diffusion images (*b* = 0 s/mm^2^) were co-registered to the corresponding T1-weighted images with an affine transformation. Meanwhile, the T1-weighted images were non-linearly transformed to the Montreal Neurological Institute (MNI) space using the ICBM-152 brain template. Thereafter, these two transformations were inversed and combined into one transformation, which was applied to wrap the AAL from MNI space to the native diffusion space of each participant. Finally, the 90 × 90 symmetric connectivity matrix for each participant was constructed by calculating the mean FA values of streamlines that connect each node pair.

### Structural network analyses

The global and regional network metrics, as well as the modular metrics, were calculated to characterize the topological properties of altered structural networks. All the following network metrics were calculated using GRETNA.^3^

### The global and regional network metrics

The global metrics calculated in our study consisted of global efficiency (E_*glob*_), local efficiency (E_*loc*_), cluster coefficient (Cp), shortest path length (Lp), normalized cluster coefficient (γ), normalized characteristic path length (λ), and small-worldness (σ). For regional properties, the following two nodal metrics were considered: nodal efficiency (NE) and degree centrality (DC). The definition and interpretation of these network metrics can be referred to Rubinov and Sporns (2010).

To avoid both spurious connections and bias of a single sparse threshold, the area under the curve (AUC) under sparsity, ranging from 27 to 40% with an interval of 0.5% for each global and regional measures, was calculated for the following statistical analysis.

### The modular metrics

**FIGURE 2:**
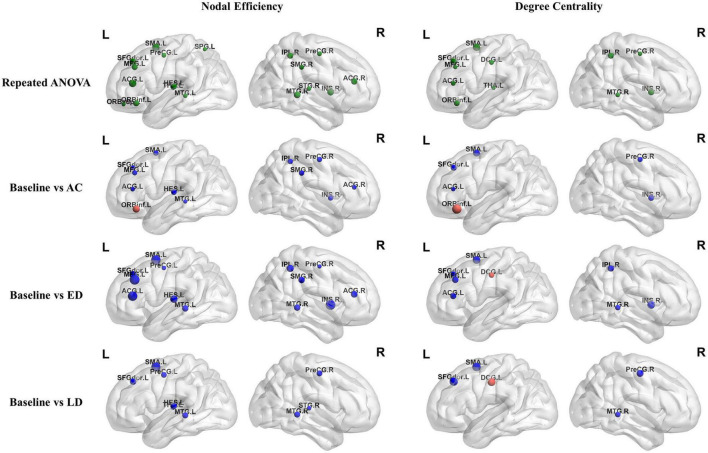
The within-module and between-module connectivity strengths analysis of the four modules used. L, left hemisphere; R, right hemisphere; AC, post-RT-AC; ED, post-RT-ED; LD, post-RT-LD; **p < 0.01.

With regards to the modularity analysis, the total number of modules and the associated module membership of nodes were optimized by maximizing modularity Q, the detailed definition and interpretation of which can be referred to Newman, 2006. Particularly, the Louvain algorithm in the Brain Connectivity toolbox^4^ was used to optimize the *Q*-value under varying sparsity, ranging from 0.05 to 0.3. Generally, a *Q*-value > 0.3 indicated a strong modular structure (Fortunato and Barthelemy, 2007; Hilger et al., 2017). Given that the number of modules and their membership varied across different sparsity, sparsity was fixed at 0.1 (*Q* = 0.39) to obtain a quite reasonable and consensus modularity partition, as shown in Figure 2. Thereafter, to assess the modular segregation, the within-module connectivity and between-module connectivity, which are defined as the strengths of edges within a single module and between a pair of modules, respectively, were calculated.

### Statistical analysis

The one-way repeated measures analysis of variance (ANOVA) was used to compare four groups at the baseline, and three follow-up stages (post-RT-AC, post-RT-ED, and post-RT-LD) after RT for all of these metrics. All the measured data satisfied the assumptions of normality and homogeneous variance. The assumption of sphericity was violated for the regional network metrics in several brain regions, where the Greenhouse–Geisser method was used to correct the sphericity. Thereafter, a *post-hoc* analysis (multiple comparisons) was performed by using paired *t*-test to compare each pair within the four groups. When the differences between the paired observations did not follow a normal probability distribution, Wilcoxon Signed-Rank test, which is a non-parametric equivalent of the paired *t*-test, was used instead. Finally, false discovery rate (FDR) correction was used for multiple comparisons.

In addition, dose-response analysis was performed by calculating the Spearman’s rank correlation coefficient (*r*-value) of the association between the abnormal nodal metrics and the radiation dose of ipsilateral temporal lobe.

## Results

### The global analysis

**FIGURE 3:**
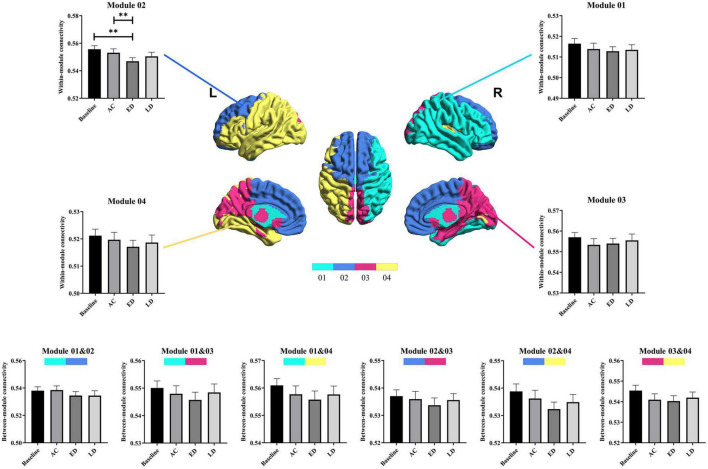
The *post-hoc* pairwise comparison results of the global network measures for four time stages (the baseline, post-RT-AC, post-RT-ED, and post-RT-LD) after false discovery rate (FDR) correction. AC, post-RT-AC; ED, post-RT-ED; LD, post-RT-LD; **p < 0.01.

Figure 3 shows the global network measures of a cohort of 35 patients at four stages, including the baseline, post-RT-AC, post-RT-ED, and post-RT-LD. All four groups exhibited small-world characteristics with λ≈1, γ > 1, and σ > 1. For the global metrics, only E_*loc*_ exhibited a significant difference among four groups after FDR correction. Specifically, E_*loc*_ significantly decreased at post-RT-ED and post-RT-LD, compared to baseline. In addition, E_*loc*_ showed a recovering tendency at post-RT-LD, although no significant difference existed between post-RT-ED and post-RT-LD. Moreover, E_*glob*_ showed a tendency of progressive decrease at post-RT-AC and post-RT-ED and a partial recovery at post-RT-LD. Similarly, the small-world coefficient, sigma (σ), showed a tendency of increase at post-RT-ED and a partial recovery at post-RT-LD. The statistical results are shown in Table 1.

**TABLE 1:** The statistical results of the global network measures for four time stages.

Global network measures		Baseline vs. AC	Baseline vs. ED	Baseline vs. LD	AC vs. ED	AC vs. LD	ED vs. LD	Rep_ANOVA
E_glob_	uncorr_p	0.1255	0.0094**	0.1170	0.2801	0.9243	0.3180	0.0608
	corr_p	0.2510	0.0564	0.3510	0.4202	0.9243	0.3815	
E_loc_	uncorr_p	0.1165	0.0033**	0.0069**	0.0331*	0.3845	0.2379	0.0049**
	corr_p	0.1748	0.0197*	0.0206*	0.0661	0.3845	0.2855	
Cp	uncorr_p	1	0.0918	0.2136	0.3200	0.2538	0.7941	0.3659
	corr_p	1	0.5506	0.6408	0.4800	0.5077	0.9530	
Lp	uncorr_p	0.1083	0.0089**	0.1189	0.3641	0.9980	0.3383	0.0624
	corr_p	0.3249	0.0532	0.2379	0.4369	0.9980	0.5074	
γ	uncorr_p	0.9922	0.0512	0.4978	0.0506	0.5376	0.3965	0.3075
	corr_p	0.9922	0.1535	0.7467	0.3034	0.6451	0.7931	
λ	uncorr_p	0.2689	0.8708	0.4245	0.3451	0.5554	0.3566	0.6038
	corr_p	1	0.8708	0.6367	1	0.6665	0.7131	
σ	uncorr_p	0.8880	0.0427*	0.5219	0.0291**	0.4935	0.3397	0.2363
	corr_p	0.8880	0.1280	0.6263	0.1748	0.7402	0.6793	

The statistical results with *p*-values of the global network measures of 35 patients for four groups (the baseline, post-RT-AC, post-RT-ED, and post-RT-LD). E_glob_, global efficiency; E_loc_, local efficiency; Cp, cluster coefficient; Lp, shortest path length; γ, normalized cluster coefficient; λ, normalized characteristic path length; σ, small-worldness; uncorr_p, uncorrected *p*-value; corr_ p, corrected *p*-value with the false discovery rate (FDR) correction; Rep_ANOVA, one-way repeated measures analysis of variance; AC, post-RT-AC; ED, post-RT-ED; LD, post-RT-LD; *p < 0.05; **p < 0.01.

### The regional analysis

**FIGURE 4:**
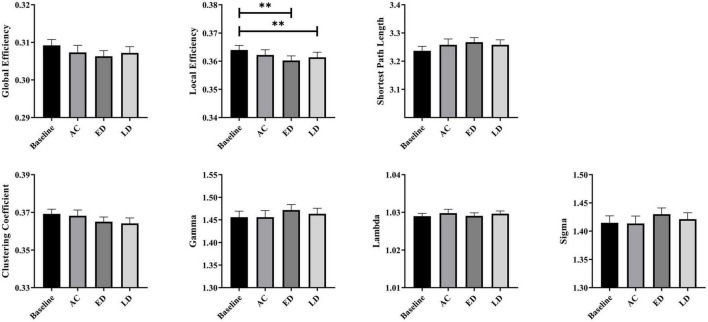
The regions with significantly different nodal efficiency and degree centrality across four stages by repeated measures ANOVA and for post-hoc pairwise comparisons between the baseline and three following post-RT stages (post-RT-AC, post-RT-ED, and post-RT-LD). The full names of the AAL atlas regions with corresponding abbreviations are listed in Supplementary Table 1. L, left hemisphere; R, right hemisphere; AC, post-RT-AC; ED, post-RT-ED; LD, post-RT-LD; Repeated ANOVA, one-way repeated measures analysis of variance.

Figure 4 shows the significantly altered regions for nodal efficiency and degree centrality by repeated measures ANOVA among four groups and by *post-hoc* pairwise comparison between the baseline and three post-RT stages (post-RT-AC, post-RT-ED, and post-RT-LD). The statistical results are shown in Table 2, and the relevant information of 90 regions from the AAL atlas together with corresponding abbreviations are listed in Supplementary Table 1.

**TABLE 2:** The statistical results of regional analysis for nodal efficiency and degree centrality.

Regions	NE				DC			
	Rep_ANOVA	Baseline vs. AC	Baseline vs. ED	Baseline vs. LD	Rep_ANOVA	Baseline vs. AC	Baseline vs. ED	Baseline vs. LD
ORBinf.L	0.0106*	0.0043**	0.2303	0.1277	0.0140*	0.0008**	0.2102	0.0788
SFGdor.L	0.0041**	0.0220*	0.0034**	0.0076**	0.0048**	0.0099**	0.0074**	0.0018**
MFG.L	0.0111*	0.0208*	0.0006**	0.1581	0.0416*	0.0827	0.0076**	0.6431
ACG.L	0.0040**	0.0323*	0.0008**	0.1971	0.0294*	0.0399*	0.0107*	0.5435
SMA.L	0.0041**	0.0160*	0.0008**	0.0010**	0.0043**	0.0056**	0.0031**	0.0017**
THA.L	0.0427*	0.5560	0.6878	0.0317*	0.0479*	0.0967	0.7270	0.1639
PreCG.R	0.0289*	0.0189*	0.0430*	0.0128*	0.0386*	0.0212*	0.0575	0.0055**
INS.R	0.0068**	0.0177*	0.0006**	0.0745	0.0093**	0.0120*	0.0031**	0.0924
MTG.R	0.0073**	0.1240	0.0088**	0.0114*	0.0299*	0.2303	0.0173*	0.0155*
IPL.R	0.0116*	0.0182*	0.0051**	0.8055	0.0129*	0.1522	0.0090**	0.2650
ORBsup.L	0.0450*	0.3321	0.0619	0.7183	−	−	−	−
HES.L	0.0109*	0.0148*	0.0039**	0.0094**	−	−	−	−
MTG.L	0.0338*	0.0427*	0.0080**	0.0109*	−	−	−	−
SPG.L	0.0437*	0.0898	0.0637	0.6225	−	−	−	−
PreCG.L	0.0289*	0.0579	0.0322*	0.0139*	−	−	−	−
ACG.R	0.0111*	0.0319*	0.0071**	0.0542	−	−	−	−
STG.R	0.0326*	0.4946	0.1718	0.0333*	−	−	−	−
SMG.R	0.0279*	0.0172*	0.0082**	0.0692	−	−	−	−
DCG.L	−	−	−	−	0.0265*	0.2945	0.0168*	0.0024**

The *post-hoc* comparison statistical results between the baseline and three post-RT time points (post-RT-AC, post-RT-ED, and post-RT-LD) for regions with significantly different nodal efficiency and degree centrality across four time points. Eighteen regions with significant difference among four groups for the nodal efficiency and 11 regions for the degree centrality were present. The values in the table are the corrected *p*-values with the false discovery rate (FDR) correction. The full names of the AAL atlas regions with corresponding abbreviations are listed in Supplementary Table 1. NE, nodal efficiency; DC, degree centrality; Rep_ANOVA, one-way repeated measures analysis of variance; AC, post-RT-AC; ED, post-RT-ED; LD, post-RT-LD; *p < 0.05; **p < 0.01.

For nodal efficiency, 18 regions had significant differences among the four groups. They were found in the prefrontal (ORBinf.L, ORBsup.L, SFGdor.L, MFG.L, ACG.L&R), temporal (HES.L, MTG.L&R, STG.R), parietal (IPL.R, SMG.R, SPG.L), frontal (SMA.L, PreCG.L&R), and subcortical (INS.R, THA.L) lobes. In these significantly altered nodes, only ORBinf.L increased at post-RT-AC, when compared to the baseline. Except for ORBinf.L, the nodal efficiency decreased at the follow-up stages after RT. In most regions with significantly decreased nodal efficiency after RT, the significant difference between baseline and post-RT-ED was larger than that between baseline and post-RT-AC and between baseline and post-RT-LD. This finding implied that the efficiency of parallel information transfer of the node first decreased and then recovered to a certain extent over time after RT.

The degree centrality exhibited significant difference among the four stages in the prefrontal (ORBinf.L, SFGdor.L, MFG.L, ACG.L), frontal (SMA.L, PreCG.R, DCG.L), temporal (MTG.R), parietal (IPL.R), and subcortical (INS.R, THA.L) lobes. For the ACG.L and INS.R, the degree centrality showed a significant and sustained decrease at post-RT-AC and post-RT-ED, and exhibited a full recovery at post-RT-LD, when compared to baseline. For the MFG.L and IPL.R, the degree centrality began to decrease at post-RT-ED and exhibited a full recovery at post-RT-LD. The PreCG.R, MTG.R, SMA.L, and SFGdor.L showed a significant and sustained decrease within 1 year after RT without recovering trend. In addition, the degree centrality showed a significant increase at post-RT-AC in the ORBinf.L and a significant and sustained increase at post-RT-ED and post-RT-LD in the DCG.L.

### The modularity analysis

Four modules were identified according to the mean network matrix of whole patients at baseline. The detailed information of four modular networks are summarized in Table 3. The modularity analysis results are shown in Figure 2.

**TABLE 3:** The detailed information on four modular networks.

Modules	AAL atlas regions		
Module 01	PreCG.R	LING.R	SMG.R
	MFG.R	SOG.R	ANG.R
	ORBmid.R	MOG.R	PUT.R
	IFGoperc.R	IOG.R	STG.R
	IFGtriang.R	FFG.R	TPOsup.R
	ORBinf.R	PoCG.R	MTG.R
	ROL.R	SPG.R	TPOmid.R
	INS.R	IPL.R	ITG.R
Module 02	SFGdor.L	OLF.L	REC.R
	SFGdor.R	OLF.R	ACG.L
	ORBsup.R	SFGmed.L	ACG.R
	MFG.L	SFGmed.R	DCG.L
	IFGoperc.L	ORBsupmed.L	DCG.R
	SMA.L	ORBsupmed.R	PCL.L
	SMA.R	REC.L	PCL.R
Module 03	PCG.L	CAL.L	CAU.L
	PCG.R	CAL.R	CAU.R
	HIP.L	CUN.L	PAL.R
	HIP.R	CUN.R	THA.L
	PHG.L	SOG.L	THA.R
	PHG.R	PCUN.L	PCUN.R
	AMYG.R	HES.L	HES.R
Module 04	PreCG.L	LING.L	ANG.L
	ORBsup.L	MOG.L	PUT.L
	ORBmid.L	IOG.L	PAL.L
	IFGtriang.L	FFG.L	STG.L
	ORBinf.L	PoCG.L	TPOsup.L
	ROL.L	SPG.L	MTG.L
	INS.L	IPL.L	TPOmid.L
	AMYG.L	SMG.L	ITG.L

The AAL atlas regions included in the four modular networks. The full names of the AAL atlas regions with corresponding abbreviations are listed in Supplementary Table 1.

For the within-module connectivity strengths, only module 2 showed a statistically significant difference among the four stages. The module 2 comprises 21 regions, including the prefrontal lobe, frontal lobe, and parts of the parietal lobe. It has a high overlap with the default mode network (DMN), and is related to the normal cognitive and emotional functions. Specifically, the connectivity strength within module 2 at the post-RT-ED was significantly lower than those at baseline and post-RT-AC. In addition, the within-module connectivity strengths for each module showed a tendency of progressive decrease at post-RT-AC and post-RT-ED and then exhibited a recovering trend at the post-RT-LD, when compared to baseline, but no significant difference was found.

For between-module connectivity strengths, no significant differences were found for each pair of modules. However, all the between-module connection strengths, except for that between modules 1 and 3, showed a tendency of sustained and progressive decrease at post-RT-AC and post-RT-ED and then exhibited a recovering trend at post-RT-LD, when compared to baseline.

### Dose-correlation analysis

**FIGURE 5:**
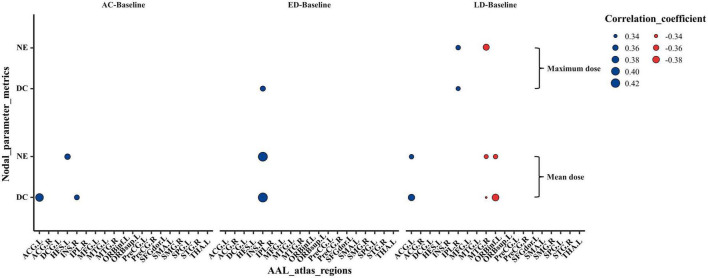
The Spearman’s correlations between the abnormal nodal metrics (NE and DC) and the mean as well as maximum irradiation dose of ipsilateral temporal lobe. The Spearman’s rank correlation coefficients (*r*-value) at the significance level of *p* < 0.05 were shown. The size of dots indicated the strength of the correlations and the color of dots indicated the orientation of the correlations (Blue: positive correlation, Red: negative correlation). NE, nodal efficiency; DC, degree centrality; AC, post-RT-AC; ED, post-RT-ED; LD, post-RT-LD.

Figure 5 shows the correlations between the abnormal nodal parameter metrics (NE and DC) and the radiation dose of ipsilateral temporal lobe. The mean and/or maximum doses of ipsilateral temporal lobe were correlated with the changed NE and DC in several regions which were distributed in temporal, subcortical, prefrontal and parietal. In brief, the changed NE and DC were positively correlated with mean and/or maximum dose at ACG.L, INS.R, HES.L, and IPL.R, and negatively correlated at ORBinf.L and MTG.R. In addition, more brain regions exhibited a significant dose correlation with NE and DC at late-delayed period; more brain regions were correlated to the mean dose than the maximum dose. The spearman’s correlations (*r*-values) with significant differences (*p*-values) for the mean dose and the maximum dose were shown in Supplementary Tables 2, 3, respectively.

## Discussion

To our knowledge, this study is the first longitudinal cohort study to monitor the RT-induced alterations of brain structural network in patients with NPC after RT. The DTI probabilistic tractography and graph theoretical approach were used to assess RT-related brain changes at the global, local, and modular levels; the following findings were obtained: (1) E_loc_ shows a significant difference among four stages. Both E_glob_ and E_loc_ show a tendency of progressive decrease at post-RT-AC and post-RT-ED and a partial recovery at post-RT-LD. (2) Except for the ORRinf.L and DCG.L, all other regions exhibited significant reductions in the nodal efficiency and degree centrality at post-RT-AC and post-RT-ED, and most of these regions showed a partial or full recovery at post-RT-LD. (3) The within-module connectivity strength of modular 2 exhibited significant and progressive decrease at post-RT-AC and post-RT-ED, compared to baseline, and showed a partially recovering trend at post-RT-LD. All the within- and between- module connectivity strengths, except for that between modules 1 and 3, showed a tendency of sustained and progressive decrease at post-RT-AC and post-RT-ED. Thereafter, a recovering trend at post-RT-LD was exhibited. All these findings imply that the brain injures begin at post-RT-AC, are aggravated at post-RT-ED, and undergo brain reorganization at the post-RT-LD. (4) The temporal irradiation dose was significantly correlated to the altered nodal parameters at the temporal (MTG.R and HES.L), subcortical (INS.R), prefrontal (ORBinf.L and ACG.L) and parietal (IPL.R), which suggests that these regions were more sensitive to dose and should be paid more attention during RT treatment plans.

The global network analysis revealed that the structural brain network possessed small-world properties (λ≈1, γ > 1, and σ > 1), at baseline and all three follow-up stages (post-RT-AC, post-RT-ED, and post-RT-LD). These results illustrate that the small-world networks are relatively robust to the changes of brain white matter (He et al., 2009; Colombo, 2013; Xu et al., 2017). For the presented global measures, only E_loc_ had statistically significant difference among the four groups. E_loc_ represents the efficiency of information exchange within a local subnetwork or among adjacent regions (Jiang et al., 2020). Reduced E_loc_ in a structural brain network may arise from the RT-associated injures of the fiber tracks (e.g., demyelination and axonal damage) (Nazem-Zadeh et al., 2012; Qiu et al., 2021). The results of significant decrease in E_loc_ at post-RT-ED and post-RT-LD were compatible with findings of prior fMRI studies reporting lower efficiency of information transfer after RT (Ding et al., 2018; Leng et al., 2021). Notably a significant decrease in E_loc_ firstly occurred 6 months after RT in our structural network study, later than the significant abnormalities in global properties of functional networks (<6 months) (Leng et al., 2021). These findings were plausible because brain function might be more vulnerable or sensitive to attack (Karim et al., 2017). In addition, both E_glob_ and E_loc_ showed a tendency of progressive decrease at post-RT-AC and post-RT-ED and partial recovery at post-RT-LD, although this trend was not statistically significant. These inconspicuous changes in trend of E_glob_ and E_loc_ may explain the inconsistent and unstable results from previous studies. Some DTI studies found a gradual and irreversible white matter damage (Nagesh et al., 2008; Welzel et al., 2008; Ding et al., 2018), whereas other groups found that the DTI metrics decreased in the early stage but partially recovered later (Wang et al., 2012; Xiong et al., 2013; Chen et al., 2015).

The significant alteration of the nodal parameters (nodal efficiency and degree centrality) among the four stages was mainly located in the temporal, frontal, prefrontal, parietal, and subcortical regions. Most of these regions showed a progressive decrease during 0–6 months post-RT and a partial or full recovery 12 months post-RT. This result may indicate that the structural brain reorganization mainly occurred in the late-delay stage, which is generally consistent with findings of previous studies (Wang et al., 2012; Xiong et al., 2013; Duan et al., 2016; Chen et al., 2020). However, some regions, including MTG.L&R, HES.L, PreCG.L&R, SFGdor.L, and SMA.L, exhibited a sustained decrease without recovering tendency within 1 year after RT, which may be due to two reasons: vulnerability of these regions to radiation causing an irreversible damage and the need of these regions for a longer recovery time (>12 months), which cloud not be observed in this 1-year longitudinal study after RT. The bilateral temporal lobes, including MTG.L&R and HES.L, exhibited decreased nodal parameters without recovering tendency over the time after RT. This observation was not surprising because the temporal lobe is often inside the target volume and inevitably receives high-dose radiation and may suffer from severe injury. Late-delayed temporal injuries have been well documented as irreversible, and sometimes presented as necrosis of temporal lobes on routine medical imaging examinations (Mao et al., 2014; Lv et al., 2019). Additionally, the nodal parameters showed significant changes in the prefrontal, frontal, and parietal regions, which were outside the irradiation field. Previous TBSS analysis (Duan et al., 2016) revealed that the fractional anisotropy values were significantly lower in the frontal, parietal, and occipital WM after RT. A previous VBM study found a reduced GM volume in the frontal and parietal cortices (Lv et al., 2014). Altogether, the changes in nodal parameters in the prefrontal, frontal, and parietal regions may arise from the degeneration of associated white matter fibers or radiation-induced disruption of the blood brain barrier (BBB) (van Vulpen et al., 2002). Notably, the increased nodal parameters in the ORBinf.L and DCG.L might act as a compensatory mechanism that maintains normal cognitive function. The subcortical regions, including the THA.L and INS.R, exhibited a different changing pattern, when compared with baseline. Specifically, the INS.R shows a “decrease-decrease-recover” pattern after RT for both nodal efficiency and degree centrality, whereas THA.L begins to decrease 12 months post-RT (post-RT-LD) for nodal efficiency. The alteration of structural brain network in the insular and thalamus is probable, given that both regions are parts of the paralimbic system that are sensitive to irradiation. In addition, these findings are consistent with those of previous studies (Ding et al., 2018; Qiu et al., 2018; Yang et al., 2019; Zhang et al., 2020; Nan et al., 2022), which reported functional and/or morphological changes in the thalamus and insula.

The dose-correlation analysis shows the nodal parameters (NE and DC) had a positive correlation with temporal dose at ACG.L, INS.R, HES.L, and IPL.R, which may be due to the compensatory change in structural brain network that interconnects these regions. Whereas the nodal parameters had a negative correlation with temporal dose at ORBinf.L and MTG.R, indicating that a higher dose reduces the information transfer efficiency to these regions. In addition, through acute reaction stage to late-delayed stage, the number of significant dose-correlation brain regions increased. This finding suggests that the dose effect on brain change is more notable at the late-delayed stage. Furthermore, some brain regions were correlated to the mean dose and/or maximum dose which illustrates that both the mean dose and the maximum dose should be considered for the protection of normal organs.

This study explored the changed patterns of structural modularity over time after RT in patients with NPC. We found that the connectivity strength within module 2 at the post-RT-ED were significantly weaker than those at baseline and post-RT-AC, indicating radiation-induced disruption of topological organization of module 2. The module 2 mainly includes the prefrontal lobe, frontal lobe, and parts of the parietal lobe. The areas of module 2 and the DMN have a large overlap, and the DMN is associated with normal cognition and emotion (Alves et al., 2019). Moreover, the module 2 includes the medial prefrontal lobe, whereas module 4 includes the left side of the temporal lobe and parietal lobe. Several fiber bundles run between the medial prefrontal lobe and temporal lobe, which is highly related to memory processing (Vertes et al., 2007). The decreased connectivity strengths within module 2 and between modules 2 and 4 at the acute- and early delayed stages may be due to the damage of axonal fiber tracts between the medial prefrontal lobe and temporal lobe. These findings support the psychological disorders, cognitive dysfunction, and mood disorders commonly found in patients with NPC after RT (Tang et al., 2012; Mo et al., 2014; Wu et al., 2014). In addition, a “decrease-decrease-partially recovery” pattern was observed for the connectivity strengths within each module and between each pair of modules, although no significant alterations were found except for connectivity strengths within module 2. These observed results were roughly consistent with the findings on nodal parameters, further implying that the brain undergoes recovery and reorganization of structure to a certain extent at the late-delayed stage.

Despite the merits of this longitudinal study, several limitations were identified. First, the 1-year follow-up was insufficient to monitor all the dynamic changes in structural network properties after RT over time. A longer period, ranging over several years, should be considered to understand whether the injured structural network topology will eventually recover to “baseline” with time. Second, this study included 35 patients with NPC; this sample size was not large enough. A larger cohort size of patients with NPC is needed to provide more reliable statistical results and to accurately reveal the dynamic changing pattern of structural brain network after RT. Third, the relationship between the alterations in structural brain network and cognitive decline were not explored because of incomplete neurocognitive outcomes.

## Conclusion

The follow-up data were used to track the dynamic changes in structural brain network after RT in patients with NPC. Our study found that the radiation-induced alterations in topological properties mainly began at the acute reaction stage, were aggravated at the early delayed stage, and then partially recovered at the late-delayed stage. The dynamic change patterns of topological properties facilitate to better understand how the radiation-induced brain injuries evolves over time and the early detection of radiation-induced changes in normal-appearing brain tissue to improve patient survival. In addition, a dose-correlation alteration was found in the temporal (MTG.R and HES.L), subcortical (INS.R), prefrontal (ORBinf.L and ACG.L), and parietal (IPL.R), indicating that these regions were more sensitive to dose and should be mainly considered in radiotherapy treatment plan.

## Data availability statement

The raw data supporting the conclusions of this article will be made available by the authors, without undue reservation.

## Ethics statement

The studies involving human participants were reviewed and approved by the Sun Yat-sen University Cancer Center. The patients/participants provided their written informed consent to participate in this study.

## Author contributions

YY, YF, GF, and JL contributed to design of the study and data collection. XZ, JPa, PX, and YL were responsible for experimental implementation. YL, PX, CY, JPe, and XL performed the data analysis. XZ, JPa, GF, YY, and YF contributed to the manuscript writing. All authors read and approved the published version of the manuscript.

## Footnotes

1
https://www.mrtrix.org

2
https://fsl.fmrib.ox.ac.uk

3
http://www.nitrc.org/projects/gretna/

4
http://www.brain-connectivity-toolbox.net/
