# Supplementary material for: Structural network alterations in patients with nasopharyngeal carcinoma after radiotherapy: A 1-year longitudinal study
Source: Front Neurosci. 2022 Nov 17;16:1059320. doi: 10.3389/fnins.2022.1059320 (PMC9712970; doi:10.3389/fnins.2022.1059320)
Supplement: Supplementary file 1 [file Table_1.PDF]

Table S1. The information of the AAL atlas regions

Labels	Regions	Abbreviation	Anatomical classification	Labels	Regions	Abbreviation	Anatomical classification
1, 2	Precental gyrus	PreCG	Frontal	47, 48	Lingual gyrus	LING	Occipital
3, 4	Superior frontal gyrus, dorsolateral	SFGdor	Prefontal	49, 50	Superior occipital gyrus	SOG	Occipital
5, 6	Superior frontal gyrus, orbital part	ORBsup	Prefontal	51, 52	Middle occipital gyrus	MOG	Occipital
7, 8	Middle frontal gyrus	MFG	Prefontal	53, 54	Inferior occipital gyrus	IOG	Occipital
9, 10	Middle frontal gyrus, orbital part	ORBmid	Prefontal	55, 56	Fusiform gyrus	FFG	Temporal
11, 12	Inferior frontal gyrus, opercular part	IFGoperc	Prefontal	57, 58	Postcentral gyrus	PoCG	Parietal
13, 14	Inferior frontal gyrus, triangular part	IFGtriang	Prefontal	59, 60	Superior parietal gyrus	SPG	Parietal
15, 16	Inferior frontal gyrus, orbital part	ORBinf	Prefontal	61, 62	Inferior parietal, but supramarginal and angular gyri	IPL	Parietal
17, 18	Rolandic operculum	ROL	Frontal				
19, 20	Supplementary motor area	SMA	Frontal	63, 64	Supramarginal gyrus	SMG	Parietal
21, 22	Olfactory cortex	OLF	Prefontal	65, 66	Angular gyrus	ANG	Parietal
23, 24	Superior frontal gyrus, medial	SFGmed	Prefontal	67, 68	Precuneus	PCUN	Parietal
25, 26	Superior frontal gyrus, medial orbital	ORBsupmed	Prefontal	69, 70	Paracentral lobule	PCL	Parietal
27, 28	Gyrus rectus	REC	Prefontal	71, 72	Caudate nucleus	CAU	Subcortical
29, 30	Insula	INS	Subcortical	73, 74	Lenticular nucleus, putamen	PUT	Subcortical
31, 32	Anterior cingulate and paracingulate gyri	ACG	Prefontal	75, 76	Lenticular nucleus, pallidum	PAL	Subcortical
33, 34	Median cingulate and paracingulate gyri	DCG	Frontal	77, 78	Thalamus	THA	Subcortical
35, 36	Posterior cingulate gyrus	PCG	Parietal	79, 80	Heschl gyrus	HES	Temporal
37, 38	Hippocampus	HIP	Temporal	81, 82	Superior temporal gyrus	STG	Temporal
39, 40	Parahippocampal gyrus	PHG	Temporal	83, 84	Temporal pole: superior temporal gyrus	TPOsup	Temporal
41, 42	Amygdala	AMYG	Temporal	85, 86	Middle temporal gyrus	MTG	Temporal
43, 44	Calcarine fissure and surrounding cortex	CAL	Occipital	87, 88	Temporal pole: middle temporal gyrus	TPOmid	Temporal
45, 46	Cuneus	CUN	Occipital	89, 90	Inferior temporal gyrus	ITG	Temporal

Table S2. Correlations between the abnormal nodal metrics (NE and DC) and the mean irradiation dose of ipsilateral temporal lobe.

Regions	NE			DC		
	AC-Baseline	ED-Baseline	LD-Baseline	AC-Baseline	ED-Baseline	LD-Baseline
ORBinf.L			r=-0.349, p=0.040*			r=-0.379, p=0.025*
SFGdor.L						
MFG.L						
ACG.L			r=0.350, p=0.039*	r=0.402, p=0.017*		r=0.376, p=0.026*
SMA.L						
THA.L						
PreCG.R						
INS.R		r=0.429, p=0.010*		r=0.354, p=0.037*	r=0.433, p=0.009**	
MTG.R			r=-0.346, p=0.042*			r=-0.336, p=0.049*
IPL.R						
ORBsup.L				—	—	—
HES.L	r=0.362, p=0.033*			—	—	—
MTG.L				—	—	—
SPG.L				—	—	—
PreCG.L				—	—	—
ACG.R				—	—	—
STG.R				—	—	—
SMG.R				—	—	—
DCG.L	—	—	—			

Table S2. The Spearman's correlations between the abnormal nodal metrics (NE and DC) and the mean irradiation dose of ipsilateral temporal lobe. The full names of the AAL atlas regions with corresponding abbreviations are listed in Supplemental Table S1. Abbreviation; NE, nodal efficiency; DC, degree centrality; AC, post-RT-AC; ED, post-RT-ED; LD, post-RT-LD. *, p<0.05; **, p<0.01.

Table S3. Correlations between the abnormal nodal metrics (NE and DC) and the maximum irradiation dose of ipsilateral temporal lobe.

Regions	NE			DC		
	**AC-Baseline**	**ED-Baseline**	**LD-Baseline**	**AC-Baseline**	**ED- Baseline**	**LD-Baseline**
ORBinf.L						
SFGdor.L						
MFG.L						
ACG.L						
SMA.L						
THA.L						
PreCG.R						
INS.R					r=0.356, p=0.036*	
MTG.R			r=-0.369, p=0.029*			
IPL.R			r=0.350, p=0.039*			r=0.346, p=0.041*
ORBsup.L				—	—	—
HES.L				—	—	—
MTG.L				—	—	—
SPG.L				—	—	—
PreCG.L				—	—	—
ACG.R				—	—	—
STG.R				—	—	—
SMG.R				—	—	—
DCG.L	—	—	—			

Table S3. The Spearman's correlations between the abnormal nodal metrics (NE and DC) and the maximum irradiation dose of ipsilateral temporal lobe. The full names of the AAL atlas regions with corresponding abbreviations are listed in Supplemental Table S1. Abbreviation; NE, nodal efficiency; DC, degree centrality; AC, post-RT-AC; ED, post-RT-ED; LD, post-RT-LD. *, p<0.05; **, p<0.01.
